# The efficacy of Denosumab in the treatment of femoral head osteonecrosis: a retrospective comparative study

**DOI:** 10.1038/s41598-024-54685-7

**Published:** 2024-02-20

**Authors:** Jun-Ki Moon, Jinyong Park, Yisack Yoo, Jae Youn Yoon, Sunhyung Lee, Pil Whan Yoon

**Affiliations:** 1 Department of Orthopaedic Surgery, Chung-Ang University Hospital, Chung-Ang University College of Medicine, Seoul, South Korea; 2 Department of Orthopedic Surgery, Asan Medical Center, Seoul, South Korea; 3 Department of Orthopedic Surgery, Seoul Now Hospital, 372, Simin-Daero, Dongan-gu, Seoul, South Korea; 4 Department of Orthopedic Surgery, Seoul National University Boramae Medical Center, Seoul, South Korea

**Keywords:** Diseases, Musculoskeletal system, Pain

## Abstract

The present study aimed to compare clinical and radiological differences of ONFH patients who were treated with denosumab, and a control group. A total of 178 patients (272 hips) with symptomatic, nontraumatic ONFH were divided into a denosumab group (98 patients, 146 hips) and a control group (80 patients, 126 hips). Patients in the denosumab group received a 60 mg subcutaneous dose of denosumab every 6 months. For the clinical assessments, Harris hip scores (HHS), Western Ontario and McMaster Universities Osteoarthritis Index (WOMAC) were evaluated. Plain radiographs and MRI were performed before and a minimum of 1 year after administration of denosumab, which were evaluated for radiological results including femoral head collapse (≥ 2 mm) and volume change of necrotic lesion. Femoral head collapse occurred in 36 hips (24.7%) in the denosumab group, and 48 hips (38.1%) in the control group, which was statistically significant (*P* = 0.012). Twenty-three hips (15.8%) in the denosumab group and 29 hips (23%) in the control group required THA, which showed no significant difference (*P* = 0.086). At the final follow-up, 71.9% of hips in the denosumab group had a good or excellent HHS compared with 48.9% in the control group, showing a significant difference (*P* = 0.012). The denosumab group showed a significantly higher rate of necrotic lesion volume reductions compared with the control group (*P* < 0.001). Denosumab can significantly reduce the volume of necrotic lesions and prevent femoral head collapse in patients with ARCO stage I or II ONFH.

## Introduction

Osteonecrosis of the femoral head (ONFH) is a progressive degenerative disorder that often requires hip arthroplasty^1,2^. The disease is characterised by the death of mature bone cells caused by decreased vascular bone circulation to the femoral head^3^. Progressive ONFH in adults is most often treated with total hip arthroplasty (THA), which gives excellent clinical results and has a low failure rate. However, for younger patients, femoral head-preserving treatment options, such as core decompression, osteotomy, and bone grafting are primarily considered instead of THA during the early stages^4–7^. In addition, nonsurgical treatments such as the use of bisphosphonate and lipid-lowering drugs were investigated as ways of preventing femoral head collapse due to osteonecrosis^8–10^.

Pharmaceutical treatments such as bisphosphonate and statins have reportedly been effective in preventing ONFH in animal models^9,11^. However, the in vivo effects of these drugs are still controversial. A recent randomised controlled study reported that bisphosphonate did not prevent femoral head collapse or reduce the need for THAs^8^. A recent meta-analytic study also noted that bisphosphonates significantly improved bone remodelling outcomes in animal models, but otherwise showed no significant clinical effects^11^. Statins were shown to reduce bone marrow adipocyte size and decrease adipogenesis and bone death in a chicken model of steroid-induced ONFH^12^. However, there is currently no high-level evidence to clinically support the use of statins.

Denosumab is a human monoclonal antibody to the receptor activator of nuclear factor-κB (RANK) ligand (RANKL) and is used to treat osteoporosis^13^. Its mechanism of action, which differs from the other antiresorptive agents such as bisphosphonates, is to bind to RANK where it inhibits the development and activity of osteoclasts and decreases bone resorption. In several recent case studies, denosumab was used to treat osteogenesis imperfecta in children and to prevent bone pain in patients with fibrous dysplasia^14–16^. Meiss et al.^17^ achieved favourable outcomes with denosumab in combination with a varus osteotomy to treat Perthes’ disease. Kim et al.^18^ reported that RANKL inhibition decreased bone resorption and femoral head deformity after ischaemic osteonecrosis in animal studies. However, clinical studies regarding whether denosumab has a positive effect on ONFH are currently lacking.

This study aimed to investigate the therapeutic potential of denosumab in the management of ONFH. Specifically, we sought to determine (1) the ability of denosumab to prevent femoral head collapse in ONFH patients, (2) differences (if any) between clinician-reported and patient-reported outcomes, and (3) differences (if any) in necrotic lesions volumes between ONFH patients treated with vs without denosumab.

## Results

Table 1 summarizes the outcomes as reported by clinicians and patients, respectively. The denosumab group exhibited a marginal improvement in mean HHS and WOMAC score at the final follow-up relative to initial mean scores (P = 0.105 and P = 0.086, respectively). Conversely, these scores significantly worsened in the control group (P < 0.001 and P < 0.001, respectively). There were significant differences in the mean HHS and WOMAC at the final follow-up between both groups (P = 0.008 and P < 0.001, respectively).

**Table 1:** Comparison of clinical outcomes between the denosumab and control groups.

		Denosumab group	Control group	P-value
Overall patients	No. of hips	146	126	
	HHS at the initial follow-up	65.29 ± 18.9	69.31 ± 18.48	0.154
	HHS at the final follow-up	68.29 ± 19.1	60.64 ± 19.2	0.008
	WOMAC at the initial follow-up	21.43 ± 17.28	21.68 ± 12.29	0.916
	WOMAC at the final follow-up	16.79 ± 15.95	36.05 ± 17.44	< 0.001
Patients with radiological failure	No. of hips	36 (24.7%)	48 (38.1%)	0.012
	HHS at the initial follow-up	60.1 ± 16	62.2 ± 16.9	0.676
	HHS at the final follow-up	52.9 ± 16.4	47.9 ± 14	< 0.001
	WOMAC at the initial follow-up	24.3 ± 14.3	22.5 ± 8.8	< 0.001
	WOMAC at the final follow-up	28.7 ± 19.4	48.9 ± 14.2	< 0.001
Patients without radiological failure	No. of hips	110 (75.3%)	78 (61.9%)	0.012
	HHS at the initial follow-up	67.1 ± 19.6	74.2 ± 18.1	0.051
	HHS at the final follow-up	73.7 ± 17	69.3 ± 17.4	0.097
	WOMAC at the initial follow-up	18.2 ± 13.5	21.1 ± 14.3	0.764
	WOMAC at the final follow-up	13.8 ± 12.5	27.3 ± 13.7	< 0.001
Conversion to THA	No. of hips	23 (15.8%)	29 (23%)	0.086

Femoral head collapse occurred in 36 hips (24.7%) in the denosumab group and 48 hips (38.1%) in the control group, with a significant difference between the two groups (*P* = 0.012). Among the hips with evidence of femoral head collapse, classification according to the Association Research Circulation Osseous (ARCO)^19^ revealed a distribution of two hips in stage I and 34 in stage II in the denosumab group. Conversely, the control group had nine hips classified as stage I and 39 hips as stage II (*P* = 0.105). Among patients who experienced radiological failure, the denosumab group had a worsening of the mean HHS and WOMAC score at the final follow-up relative to the initial mean scores (P = 0.031 and P = 0.121, respectively) (Table 1). Similarly, in the control group, a significant worsening in mean HHS and WOMAC score was determined at the final follow-up compared with initial mean scores (P < 0.001 and P = 0.19, respectively).

Twenty-three hips (15.8%) in the denosumab group and 29 hips (23%) in the control group required THA. There was no significant difference in the rate of THA between the two groups (*P* = 0.086). Of the 23 hips that underwent THA in the denosumab group, four were classified as stage I and 19 were classified as stage II. Of the 29 hips that underwent THA in the control group, four were classified as stage I and 25 were stage II (*P* = 0.507).

In the remaining hips without femoral head collapse, there were improvements in mean HHS and WOMAC score in the denosumab group at the final follow-up compared with the initial mean scores (P < 0.001 and P < 0.001, respectively) (Table 1). In contrast, the control group exhibited significant worsening of the mean HHS and mean WOMAC score at the final follow-up relative to initial mean scores (P < 0.001 and P < 0.001, respectively). At the final follow-up, 71.9% of hips in the denosumab group had a good or excellent HHS compared with 48.9% in the control group, showing a significant difference (P = 0.012).

**Figure 1:**
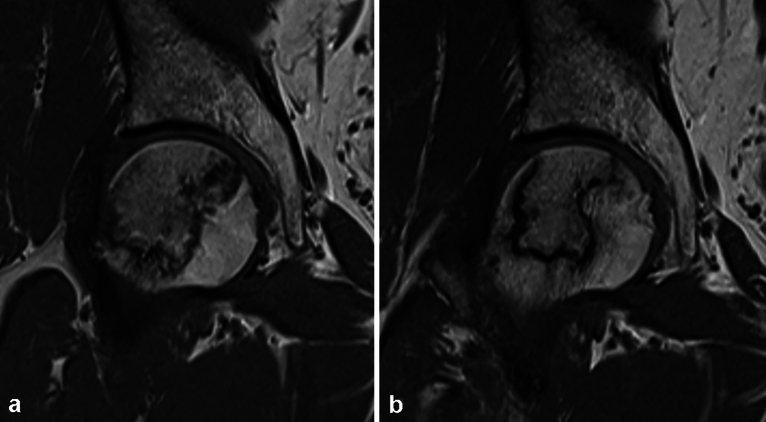
(**a**) MRI images showing a large necrotic lesion in the femoral head of a 44-year-old male patient. The calculated volume of the necrotic lesion was 23.77 cm^3^. (**b**) MRI image showing a decrease in volume of necrotic lesion 1 year after denosumab treatment. The calculated volume of the necrotic lesion was 14.33 cm^3^.

Among the remaining hips with no femoral head collapse in the denosumab group, the mean initial volume of the necrotic lesions was 13.79 (± 7.33 cm^3^), which decreased significantly to 11.87 (± 6.83 cm^3^) at the final follow-up (P < 0.001, Fig. 1). In the control group, the mean initial volume of the necrotic lesions was 11.37 (± 7.83 cm^3^), which increased slightly to 12.39 (± 7.81 cm^3^) at the final follow-up (P = 0.400). The median relative change in necrotic lesion volume was a decrease of 15.45% in the denosumab group, in contrast with a 2.45% increase in the control group. In the denosumab group, there was an increase in the volume of the necrotic lesion in 13 hips (11.6%), no change in 40 hips (35.7%), and a decrease in volume in 59 hips (52.7%). In the control group, there was an increase in the volume of the necrotic lesion in 26 hips (35.1%), no change in volume in 36 hips (48.6%), and a decrease in volume in 12 hips (16.2%). The denosumab group showed a significantly greater decrease in the volume of the necrotic lesions compared with the control group (P < 0.001).

### Survival analysis

**Figure 2:**
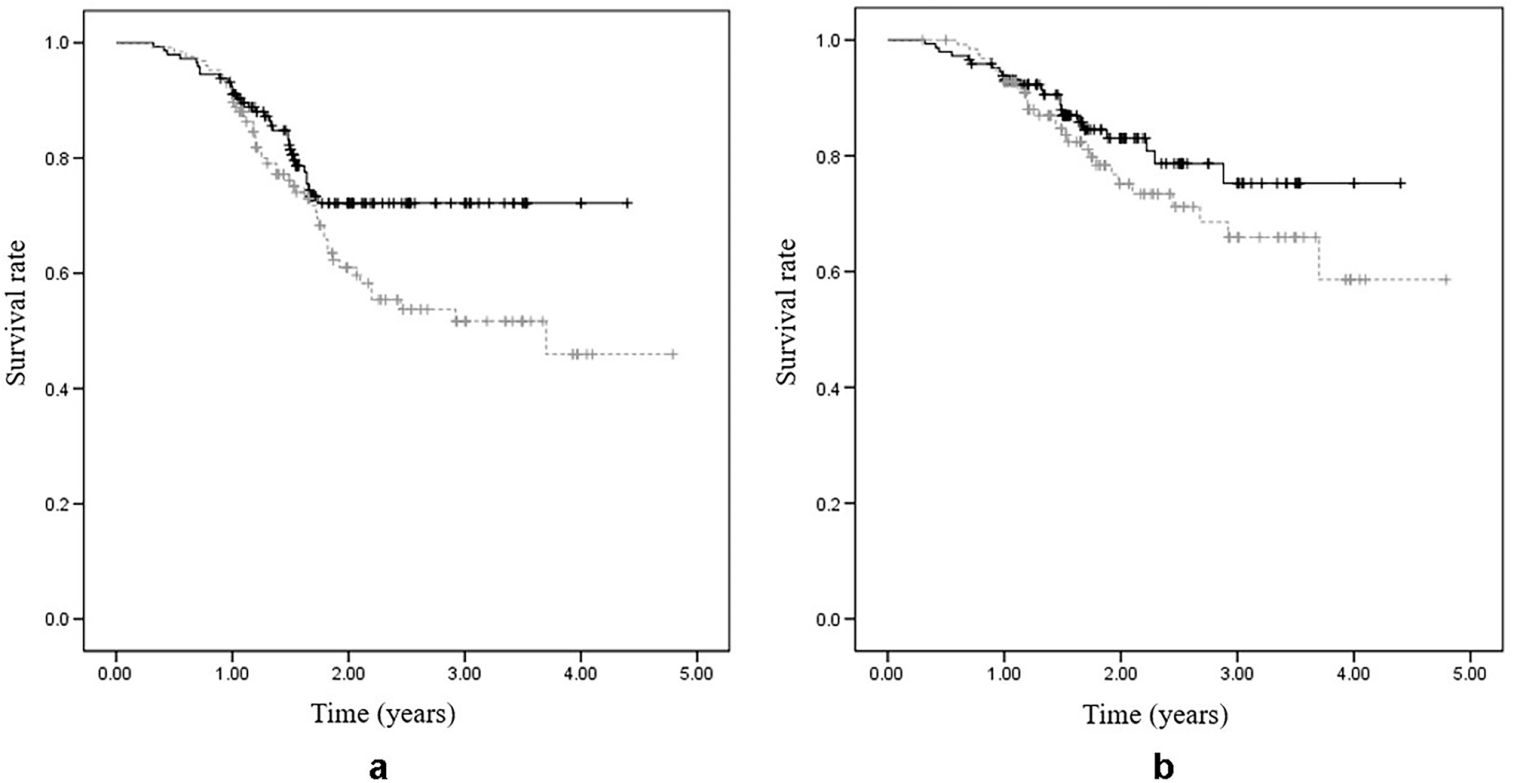
Kaplan–Meier survival analysis of (**a**) femoral head collapse and (**b**) requirement of total hip arthroplasty as the endpoints (denosumab group: black solid line, control group: grey dashed line).

After a mean follow-up period of two years (range 1–5.6), the survival rate of femoral head free from radiological failure was 72.2% (95% CI 68–76.4) in the denosumab group and 45.9% (95% CI 38.6–53.2) in the control group, and the difference was statistically significant (P = 0.034). The survival rate of the femoral head free from the requirement of THA was 75.2% (95% CI 69.7–80.7) in the denosumab group and 58.6% (95% CI 49.9–67.3) in the control group, although the difference was not statistically significant (P = 0.212, Fig. 2).

## Discussion

The authors compared the clinical and radiological outcomes of ONFH patients who had denosumab treatment and those who did not. After a minimum of 1 year’s follow-up, ONFH patients who had received denosumab treatment showed a significantly lower rate of femoral head collapse compared with the control group. Moreover, the volume of necrotic lesions decreased much more frequently after denosumab treatment, unlike in the control group. Although a few studies reported that denosumab treatment had a positive effect on ONFH patients^20^, to our knowledge, the present study is the first comparative study focusing on the rate of femoral head collapse and the change of necrotic lesion volume via MRI assessment. The important finding of this study is that necrotic lesions can be diminished after denosumab treatment in ONFH patients whose femoral head not collapsed. This suggests that denosumab can be used as an effective adjuvant therapy when combined with other hip preservation therapies for ONFH patients.

If untreated, symptoms develop in 55.9% of asymptomatic ONFH cases, then which can lead to femoral head collapse and hip joint destruction^21^. Many researchers tried to preserve the viability of the femoral head using various medical treatments (Table 2). Many animal studies have shown that bisphosphonate use significantly improved mean epiphyseal quotients, which indicate femoral head sphericity as well as better trabecular bone volume, trabecular separation, trabecular thickness, and trabecular number. However, more recent studies showed that bisphosphonate had no significant effect on the treatment of ONFH in the clinical model^8,11^. This gap between animal and human studies might be attributed to different sensitivities to bisphosphonate, different doses, and other biases.

**Table 2:** Literature review of medical treatments for osteonecrosis of the femoral head.

Authors	Treatment	Number of hips	Mean length of follow-up period (years)	Mean age at surgery (years)	Collapse rate	The rate of conversion THA
Agarwala et al.^30^	Alendronate	395 hips (294 patients)	4 (1–8)	39.1 (22–55)	28.8%	NA
Lee et al.^8^	Zoledronate vs control group	55 hips (55 patients) vs 55 hips (55 patients)	2	44 (18–73)	52.7%	34.5%
Chen et al.^31^	Alendronate vs control group	65 hips (52 patients)	2	48.4 ± 11.4	37%	12.5%
Liu et al.^19^	Denosumab vs control group	161 patients vs 209 patients	2	42.9 ± 15.1	42.2%	NA
Present study	Denosumab vs control group	146 hips (104 patients) vs 126 hips (80 patients)	2 (1–5.6)	51.6 (18–83)	24.7%	15.8%

Denosumab is now being widely used, particularly in the treatment of osteoporosis as it can inhibit osteoclastic and bone resorption in patients. Denosumab has also been suggested as a novel treatment strategy to decrease femoral head deformity after ischaemic osteonecrosis in animal models. Kim et al. reported that the animal group with RANKL inhibition showed a significantly higher epiphyseal quotient, a significant reduction in the number of osteoclasts, and better trabecular bone volume, trabecular numbers, and trabecular separation^18^. The authors also suggested RANKL inhibitors could be a potential candidate for treating paediatric bone diseases such as Legg-Calve-Perthes disease. However, clinical studies researching the effect of denosumab on ONFH are scarce.

A recent comparative study conducted by Liu et al.^20^ reported that denosumab had a positive effect on preventing femoral head collapse in patients with steroid-induced ONFH. Although the study focused on the clinical effects of denosumab on steroid-induced ONFH, the methodology for assessing radiological results was relatively unclear. In the present study, volumetric measurement using MRI clearly showed that patients taking denosumab had significantly smaller lesions than the control group. Furthermore, the mean volume of the necrotic lesions in non-collapsed ONFH significantly decreased after denosumab administration along with the collapse rate. The bone in necrotic areas contains several reversal cement lines, indicative of extensive remodelling. It also contains several empty lacunae from the loss of osteocytes and poorly vascularised calcified marrow^22^. Denosumab effectively inhibits this remodelling process in the human femoral neck^23^. Bisphosphonate, by contrast, not only inhibits this remodelling process but also impairs the onset of bone formation at remodelling sites by influencing osteoblast recruitment and decreasing the release of osteogenic factors from the osteoclasts^24^. However, denosumab preserves this modelling-based bone formation while inhibiting remodelling, which may contribute to a decrease in the necrotic volume over time. Dempster et al.^23^ reported that denosumab-treated human femoral neck showed 9.4-fold and 2.0-fold higher mean values of modelling-based bone formation in cancellous and endocortical envelopes respectively compared with controls. Furthermore, a gain in bone mineral density at non-necrotic lesions, particularly in lateral pillars after denosumab administration, might contribute to preventing the femoral head collapse^25^.

The denosumab group showed a slightly lower rate of THA than the control group, but the difference was not significant in the present study. This might be because the need for a THA depends on various economic and personal factors in addition to symptom aggravation. For example, the control group included patients who refused further treatment such as hip preserving surgery or medication. These patients might also refuse a THA despite their femoral head collapsing and their symptoms being aggravated, meaning that the rate of THA might be underestimated in the control group. A review of medical records revealed that economic reasons and personal fears were the most common reasons for THA refusal. Therefore, a prospective study that will control for these confounding factors will be necessary to clarify the effect of denosumab on THA necessity.

The present study has several limitations. A retrospective design is inherently prone to selection bias. However, the authors tried to strengthen the study with unintentionally matched comparisons. There were no significant differences in demographic and disease-related variables, including age, sex, BMI, stage, position, aetiology, and follow-up duration between both groups. Histopathologic analysis was not performed in the present study, meaning that the radiographic volumetric decrease of the necrotic area in the denosumab group could only be interpreted quantitatively. Nonetheless, using MRI to volumetrically measure necrotic lesions is known to be the most precise method to evaluate volume change in the necrotic lesions of ONFH patients^26,27^. Also, the MRI assessments were only performed at a single time point, at least 1 year after denosumab treatment or diagnosis. Therefore, the serial changes of MRI findings were not considered in this study.

In conclusion, denosumab can significantly reduce the volume of necrotic lesions and can prevent the collapse of the femoral head in patients with ARCO stage I or II ONFH. However, this study did not show a significant difference as to whether patients would require THA after receiving denosumab treatment. Therefore, a prospective controlled study will be required to verify these findings.

## Methods

The inclusion criteria were as follows: (1) patients aged > 18 years, (2) patients diagnosed with symptomatic nontraumatic ONFH with no femoral head collapse (ARCO stages I and II), and (3) patients who had completed a follow-up period of at least 1 year. Patients with metabolic bone disease, a history of previous hip disease or fractures, inflammatory joint disease, degenerative arthritis of the hip, or immunosuppressive agent use were excluded. Between February 2018 and December 2019, 104 patients (146 hips) who received denosumab subcutaneously for the treatment of ONFH met the inclusion criteria. The denosumab group was compared against a control group of 80 patients (126 hips) who were diagnosed with ONFH but did not receive denosumab. Patients in the denosumab group opted for conservative treatment over surgery, while those in the control group declined additional interventions, including surgery and denosumab administration. The demographics of the patients in both groups are presented in Table 3.

**Table 3:** Patient demographics.

	Denosumab group (n = 146)	Control group (n = 126)	P-value
Male/female (hips)	89/57	90/36	0.074
Age (range), year	51.7 (19–79)	55.1 (18–83)	0.114
Body mass index (range), kg/m^2^	23.2 (17.2–34.4)	24.1 (18.1–35)	0.552
Follow-up period (range), year	1.9 (1–3.2)	2.1 (1–5.6)	0.107
Aetiology of ONFH			0.055
Idiopathic	52 (35.6%)	35 (27.8%)	
Steroid-induced ONFH	42 (28.8%)	54 (42.9%)	
Alcohol-induced ONFH	52 (35.6%)	37 (29.4%)	
Laterality (patients)			0.021
Unilateral	62 (59.6%)	34 (42.5%)	
Bilateral	42 (40.4%)	46 (57.5%)	
ARCO classification (hips)			0.160
I	19 (13.1%)	9 (7.1%)	
II	127 (86.9%)	117 (92.9%)	
The size of necrotic lesion (cm^3^)	13.74 ± 7.35	11.37 ± 7.83	0.054
Location of lesion (hips)			0.251
Central	20	24	
Lateral	126	102	

In the denosumab group, patients received a subcutaneous 60 mg dose of denosumab every 6 months for at least 1 year. The first administration was given at the time of the initial diagnosis of ONFH by MRI. The patients also received calcium and vitamin D supplements to prevent hypocalcaemia by antiresorptive treatment. Patients in both groups were treated for pain relief with medication such as non-steroidal anti-inflammatory drugs or tramadol and were able to use a cane or crutches if required.

Two authors who did not participate in the treatment of ONFH retrospectively and independently reviewed the clinical and radiological results. Outcome data, as reported by patients and clinicians, were collected at the initial visit and the final follow-up. The Harris hip scores (HHS), Western Ontario and McMaster Universities Osteoarthritis Index (WOMAC), and requirements for secondary interventions were investigated for clinical assessments. Antero-posterior and frog-leg lateral radiographs of the affected hips were obtained using standard protocols^28^. Any progression in femoral head collapse of more than 2 mm was investigated by comparing the serial radiographs with each other. Femoral head collapse ≥ 2 mm was defined as a radiological failure, and conversion to total hip arthroplasty (THA) was regarded as a clinical failure.

A hip MRI was performed to confirm a diagnosis of ONFH in all the patients at their initial outpatient visits. At least one year after the first MRI, subsequent MRI was performed for the investigation of disease progression in 81 patients (112 hips) in the denosumab group, and 50 patients (77 hips) in the control group. The mean follow-up duration for the MRI assessments was 1.78 (range 1–2.7) years. An ONFH diagnosis was made by experienced musculoskeletal radiologists and orthopaedic surgeons. The volume of the necrotic lesion was measured on coronal T1-weighted images. A necrotic lesion was considered to be an area demarcated by the outline corresponding to a band-like hypointense lesion.

**Figure 3:**
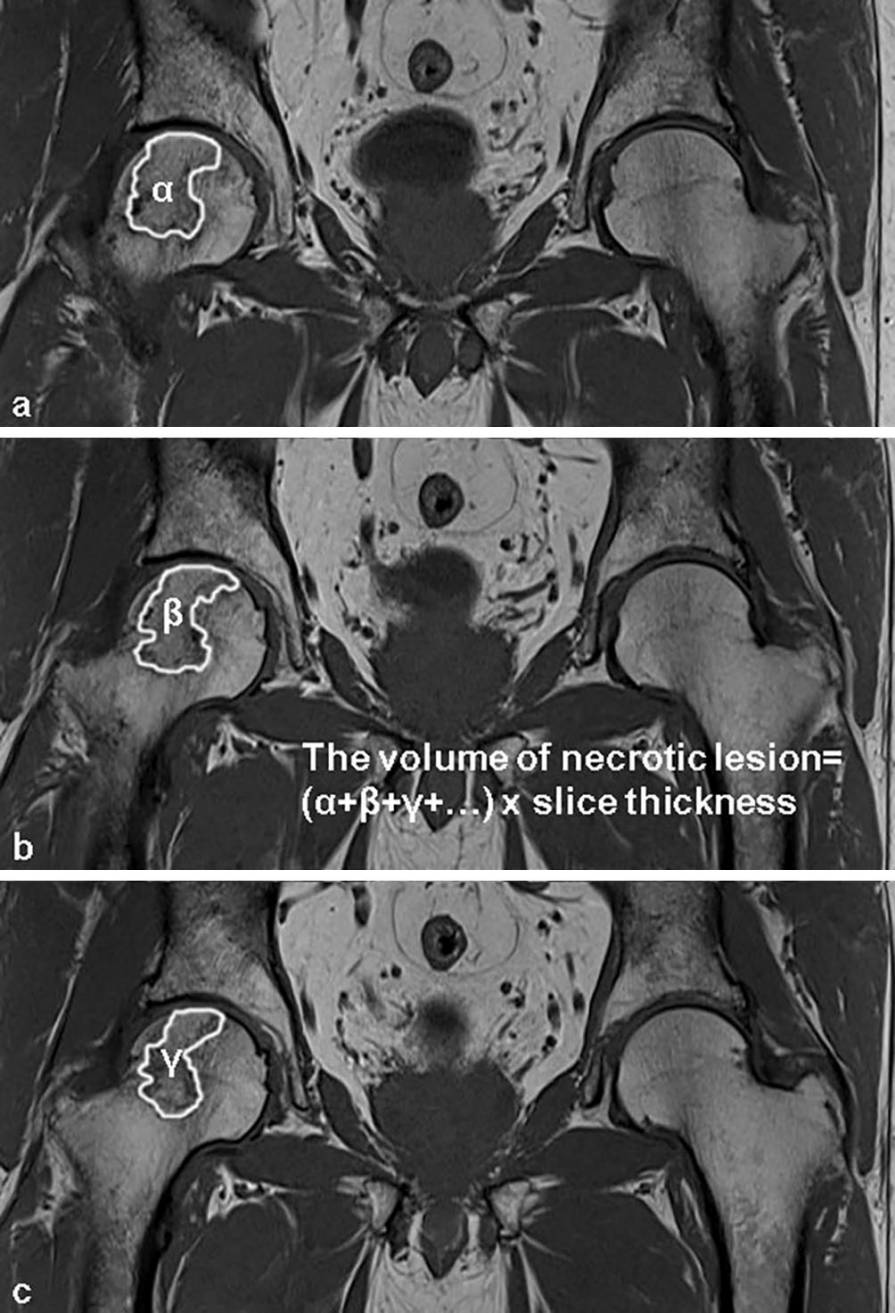
(**a–c**) Illustrations manually tracing the outline demarcation corresponding to the band-like hypointense lesion (white solid outline). The volumes of the necrotic lesions were calculated by summating the volumes obtained by multiplying the necrotic areas in each slice with the slice thickness (α: the necrotic lesion area in (**a**), β: the necrotic lesion area in (**b**), γ: the necrotic lesion area in (**c**)).

The volume of the necrotic lesions was calculated by multiplying the manually traced area of osteonecrosis by the slice thickness and summating all the slice volumes^26^ (Fig. 3). The cut-off value for changing the volume of the necrotic lesion was determined as 15% based on the study conducted by Zhao et al.^29^. A change in volume lower than 15% was regarded as “no change”. Increasing or decreasing necrotic volume by more than 15% compared with the initial MRI was considered a significant difference.

### Statistical analysis

An independent Student’s t-test was used to compare age, height, weight, follow-up duration, and the volume of the necrotic lesion. Differences in sex, aetiology, and laterality were compared using the chi-squared test, while the paired t-test was used to evaluate significant changes in HHS and WOMAC, as well as the mean volume of necrotic lesions at the different time intervals in each group. Survivorship and 95% CIs were calculated using the Kaplan–Meier method.

A priori power analysis was conducted using estimated data from a previous study reporting the incidence of femoral head collapse in ONFH patients to be as high as 40%^8^. Because RANKL inhibition showed a positive effect in decreasing bone resorption and preventing femoral head deformity after osteonecrosis in animal studies, the authors assumed that the incidence of femoral head collapse in ONFH patients treated with denosumab would be approximately 20%^18^. Using an alpha of 0.05 and a power of 0.95, a minimum sample size of 120 hips in each group was calculated to detect significant differences in the incidence of femoral head collapse between the two groups. SPSS statistical software (version 18.0; SPSS, Chicago, Illinois) was used to conduct the statistical analyses.

### Ethics approval

This study was approved by the Institutional Review Board of the Asan Medical Center (IRB number: 2022-1646). All experiments were conducted according to the guidelines of the Declaration of Helsinki.

### Informed consent

All patients gave consent to treatment according to institutional guidelines and to assessment of the clinical data and treatment outcome. This study was a retrospective trial. Therefore, the Institutional Review Board of the Asan Medical Center waived the requirement to obtain distinct written informed consent from the patients.

### Supplementary Information

Supplementary Information.

## Data Availability

Data available within the article or its Supplementary Materials.
